# *Citrus limon* Extract Loaded in Vesicular Systems for the Protection of Oral Cavity

**DOI:** 10.3390/medicines5040108

**Published:** 2018-10-14

**Authors:** Maria Manconi, Maria Letizia Manca, Carla Caddeo, Giorgia Sarais, Alessandra Palmieri, Guy D’Hallewin, Anna Maria Fadda

**Affiliations:** 1 Department of Life and Environmental Sciences, University of Cagliari, 09123 Cagliari, Italy; manconi@unica.it (M.M.); mlmanca@unica.it (M.L.M.); caddeoc@unica.it (C.C.); gsarais@unica.it (G.S.);; 2 Department of Experimental Medicine and Surgical Sciences, University of Sassari, 07100 Sassari, Italy; luca@uniss.it; 3 Institute of Science of Food Production UOS Sassari–CNR, 07100 Sassari, Italy; guy.dhallewin@gmail.com

**Keywords:** *Citrus* extract, antioxidant, antimicrobial, phospholipid vesicles, oxidative stress

## Abstract

**Background:** The nanoincorporation of the extract of *Citrus limon* (L.) Osbeck var. *pompia* into liposomes was aimed at improving its antioxidant and antibacterial effects. **Methods:** The extract of the rind of *Citrus limon* (L.) Osbeck var. *pompia* was obtained by maceration in ethanol, evaporation, and freeze-drying. The extract phytochemical fingerprint was obtained by HPLC and mass spectrometry, and it was determined that gallic acid, neohesperidin, eriocitrin, and neoeriocitrin were the most abundant components. The freeze-dried extract was loaded in liposomes, glycerosomes, and penetration-enhancer-containing vesicles prepared with propylene glycol (PG-PEVs). **Results:** Capability of the vesicles of improving efficacy of the extract in counteracting oxidative stress was studied in vitro in keratinocytes, along with antimicrobial activity against planktonic cultures of *Streptococcus mutans*, *Lactobacillus acidophilus*, and *Streptococcus sanguinis*. **Conclusion:** Results showed that the vesicles, especially glycerosomes and PG-PEVs, prevented oxidative damage and cell death, and inhibited bacterial proliferation.

## 1. Introduction

Citrus fruits have been receiving a growing interest, mainly because their use appears to be associated with reduced risk for gastro-intestinal and esophageal cancer, and cardiovascular diseases [1]. Though the citrus rind is an important source of bioactive compounds, it is commonly considered organic waste. In particular, this part of the fruit is rich in phenolic compounds that have been demonstrated to possess several valuable properties, in particular anti-inflammatory, antioxidant, and antimicrobial activities [2]. These compounds, which are secondary plant metabolites present in herbs, vegetables, and fruits, have at least one hydroxyl group linked to one or more benzene rings [3]. Thanks to the high content of phenolic compounds, several plants possess biological properties and are widely used in folk medicine. Scientific demonstration of the beneficial properties of these phytochemical-rich crops supports their exploitation for the development of new therapeutic approaches based on natural compounds.

In our previous study, the rind of *Citrus limon* (L.) Osbeck var. *pompia* Camarda [4], an ancient endemic cultivar of Sardinia (Italy), has been studied. This crop is most likely of hybrid origin and is farmed only in a few temperate areas in the North-East of the island. Because of its limited availability, only a few studies regarding extraction, characterization, and biological properties of the active compounds of *pompia* fruit can be found in the literature [5,6,7]. In a previous study, the *pompia* fruit rind was used to obtain an extract with antioxidant activity, which was incorporated in liposomes, glycerosomes, and hyalurosomes. The extract was found to have protective and regenerative properties in vitro, which were enhanced by the incorporation in phospholipid vesicles [8].

Keeping in mind these results, in the present study, the *pompia* rind was used to produce an extract rich in phenolics by maceration in ethanol. The main components of the extract were successively identified by HPLC and mass spectrometry. In order to improve the stability of the active components of the extract, several strategies have been proposed in recent years. Among them, the use of vesicular systems represents an interesting approach, as they are versatile systems capable of incorporating both hydrophilic and lipophilic active substances, which are, respectively, encapsulated in the aqueous core or incorporated in the bilayered membrane, and therefore, protected from denaturizing agents. Different studies based on liposomes have been carried out to incorporate natural substances or extracts [9,10]. Therefore, in this work the *pompia* extract was loaded in liposomes and, aiming at further improving its efficacy, in glycerosomes, and penetration-enhancer-containing vesicles (PEVs) made with propylene glycol (PG-PEVs). Glycerosomes and PG-PEVs were obtained using increasing amounts of glycerol or propylene glycol in the water phase (12.5%, 25%, 50% *v*/*v*), respectively. The physico-chemical properties and stability of the vesicles, along with their capability of protecting keratinocytes from oxidative stress induced by hydrogen peroxide, were investigated. In addition, the antibacterial activity of the formulations against cariogenic bacteria *Streptococcus mutans* and *Lactobacillus acidophilus*, and towards commensal *Streptococcus sanguinis*, were assessed.

## 2. Experimental

### 2.1. Materials

Lipoid S75 (S75), a mixture of soybean phospholipids, triglycerides, and fatty acids, was purchased from Lipoid GmbH (Ludwigshafen, Germany). Glycerol and propylene glycol were purchased from Galeno (Potenza, Italy). Ethanol and all other products were of analytical grade and were purchased from Sigma-Aldrich (Milan, Italy). Myricitrin, naringin, neoeriocitrin, and neohesperidin and all the other standards were purchased from Extrasynthese (Lyon, France). Cell culture medium and reagents (DMEM, foetal bovine serum, penicillin/streptomycin, fungizone) were purchased from Life Technologies (Monza, Italy).

### 2.2. Fruit Harvest and Extract Preparation

The fruits of *pompia* were harvested at full maturity stage in January 2015, near Cedrino River (Orosei, Sardinia). The rind of fresh fruits was peeled off, dried, and ground to obtain a powder with small particles. Aliquots of the powder (200 g) were dispersed in ethanol and transferred to a semi-automatic extractor for 5 h. The extractive dispersion was centrifuged (40 min, 1500× *g*), ethanol was removed under reduced pressure at 30 °C, and the residue dispersed in water and freeze-dried. The extract was stored under vacuum and protected from light until use. For the qualitative/quantitative determinations, the freeze-dried extract was dispersed in methanol (1:100 *w*/*v*), filtered out (0.20 µm), and analysed by HPLC-DAD.

### 2.3. HPLC-DAD Analysis

An Agilent 1100 Series HPLC (Waldbronn, Germany) equipped with a diode array detector (DAD; UV6000LP, Thermo Quest, San Josè, CA, USA) and a Spherisorb S5 ODS2 column (250 × 4.6 mm, 5 µm; Waters, Milford, MA, USA) was used. A binary mobile phase of (A) 0.1% phosphoric acid in water and (B) acetonitrile was used for multistep gradient: t_0 min_ 90% A/10% B, t_25 min_ 70% A/30% B, t_50 min_ 60% A/40% B. 50 µL of the sample was injected, and the flow rate was 1 mL/min. Spectra were recorded in the 190–600 nm range.

### 2.4. LC-MS/MS Analysis

Liquid chromatography-tandem mass spectrometry was performed using a Flexar UHPLC AS system (Perkin-Elmer, Waltham, MA, USA) equipped with a degasser, Flexar FX-10 pump, autosampler, and PE 200 column oven interfaced with an AB Sciex API4000 Q-Trap triple quadrupole linear ion trap mass spectrometer (Foster City, CA, USA). Solutions of standards (1 µg/µL in 50% methanol) were infused into the electrospray ion source at a 10 µL/min flow rate. An XSelect HSS C18 column (100 × 2.1 mm, 2.5 µm; Waters, Milford, MA, USA) was used. A binary mobile phase of (A) 0.1% formic acid in water and (B) 0.1% formic acid in acetonitrile eluted at 41 °C as follows: t_0–4 min_ isocratic 0% B (flow changes from 300 µL to 350 µL); t_4–6 min_ linear gradient 0–12% B (400 µL/min flow); t_6–12 min_ linear gradient 12–20% B (400 µL/min flow); t_16–17 min_ linear gradient 20–100% B (300 µL/min flow) was used. Analyst 1.6.2 software (AB Sciex, Foster City, CA, USA) was employed for data acquisition and processing.

### 2.5. Vesicle Preparation

Liposomes, glycerosomes, and PG-PEVs were prepared by dispersing the *pompia* extract in water or in a mixture of water/glycerol or water/propylene glycol, respectively (see Table 1).

The phospholipid (S75) and the extract were hydrated with water or the appropriate mixture overnight at room temperature, then sonicated with a Soniprep 150 (MSE Crowley, London, UK), 20 cycles, 4 s on and 4 s off (15 microns of probe amplitude). The vesicle dispersions (2 mL) were dialysed against water (4 L) at 25 °C for 3 h (with water refreshed every 30 min) to remove the non-incorporated active components, by using Spectra/Por*^®^* membranes (12–14 kDa Molecular Weight cut-off, 3 nm pore size; Spectrum Laboratories Inc., DG Breda, The Netherlands). The non-dialysed and dialysed vesicles were diluted with a DPPH (2,2-diphenyl-1-picrylhydrazyl; 40 µg/mL) methanolic solution, and the antioxidant activity was assayed as a function of the discoloration of the free radical at 517 nm [11]. The antioxidant activity (AA%) was calculated according to the following Formula (1):

AA% = [(ABS_DPPH_ − ABS_sample_)/ABS_DPPH_] × 100 (1)

The antioxidant activity found after dialysis vs. before dialysis was used to calculate the entrapment efficiency (EE%).

### 2.6. Vesicle Characterization

For the assessment of vesicle formation and morphology, the samples were stained with 1% phosphotungstic acid and observed under a JEM-1010 transmission electron microscope (TEM; Jeol Europe, Paris, France) equipped with a MegaView III camera and an AnalySIS software, at an 80 kV accelerating voltage.

For the determination of vesicle mean diameter, polydispersity index, and zeta potential, a Zetasizer nano (Malvern Instruments, Worcestershire, UK) was used, by the application of dynamic and electrophoretic light scattering.

The storage stability of the vesicles was assessed by monitoring their average size and zeta potential over 30 days at room temperature.

### 2.7. In Vitro Antioxidant Effect of the Extract

The antioxidant activity of *pompia* extract was evaluated in vitro using keratinocytes stressed with hydrogen peroxide. The cells, seeded into 96-well plates and incubated for 24 h at 37 °C in 5% CO_2_, were exposed to both hydrogen peroxide (1:30,000 dilution) and the vesicle formulations (containing 100 µg/mL of *pompia* extract) for 4 h. Untreated cells (100% viability) and cells exposed to hydrogen peroxide only were used as controls. Thereafter, the cells were gently washed with PBS, and an MTT solution [3(4,5-dimethylthiazolyl-2)-2, 5-diphenyltetrazolium bromide] (0.5 mg/mL in PBS) was added (100 µL/well) to assess cell viability [12,13]. After 3 h, the formazan crystals were solubilized in DMSO, and the absorbance was read at 570 nm using a spectrophotometer (Synergy 4, Reader BioTek Instruments, AHSI S.P.A, Bernareggio, Italy). The experiment was performed in triplicate and the results were expressed as a percentage of cell viability vs. untreated cells.

### 2.8. Determination of Antibacterial Activity

The antibacterial activity of the *pompia* formulations was assessed by performing the inhibition halo test in planktonic cultures of commensal *S. sanguinis* (ATCC 10556), and cariogenic *S. mutans* (ATCC 35668) and *L. acidophilus* (ATCC 4356). A Mueller-Hinton Agar (Sigma Aldrich, Milan, Italy) with sheep blood was used to culture the bacterial strains. The strains were aseptically transferred into test tubes containing 2 mL of saline. The turbidity of the bacterial suspensions was adjusted by using McFarland standard as a reference to reach the required number of bacteria (1–2 × 10^8^ CFU/mL). Then, 15 μL of each formulation was adsorbed on sterile 6-mm paper discs and placed in inoculated 9-cm Mueller-Hinton Petri dishes (Thermo Fisher Scientific Inc., Waltham, MA, USA) containing the specific bacterial suspension (0.1 mL). The plates were refrigerated (4 °C for 2 h) to allow the diffusion of the sample into the agar medium, and incubated for 48 h at 37 °C. The inhibitory activity, expressed as a function of the diameter of the inhibition halo, was assessed in triplicate. The positive control was represented by a paper disc without formulations to assess the growth of the bacteria (0% inhibition), and the negative control was gentamycin (10 mg/disc), a broad spectrum aminoglycoside antibiotic. The bioassays were carried out in accordance with the Clinical and Laboratory Standards Institute protocols.

### 2.9. Statistical Analysis of Data

Results are expressed as means ± standard deviations. ANOVA was used to substantiate statistical differences between sample groups, and Student’s *t*-test was used to compare two samples. *p* < 0.05 was considered statistically significant. Data analysis was carried out with the R 3.1.2 software (The R Foundation for Statistical Computing, Vienna, Austria) [14].

## 3. Results and Discussion

### 3.1. Extract Characterization

The qualitative/quantitative composition of the *pompia* extract was studied by HPLC-MS/MS, which allowed the detection of gallic acid, eriocitrin, neoeriocitrin, naringin, hesperidin, neohesperidin, and myricitrin 3-galactoside. The amount of each component, expressed as µg/mg of dry extract, is reported in Table 2.

Gallic acid was the main component of the extract (128.3 µg/mg), followed by neohespridin (76.5 µg/mg). The composition of the extract is interesting, since the main active substances detected are known to have good antioxidant activity. In a previous study, gallic acid co-loaded with resveratrol in phospholipid vesicles, showed promising antioxidant and antibacterial activities [11]. In light of these results, the antioxidant and antibacterial properties of the *pompia* rind extract were probed.

### 3.2. Vesicle Characterization

As previously reported, an extract of *pompia* rind obtained using ethanol/water (1/1) as the extractive blend, was successfully incorporated in phospholipid vesicles, which were able to improve its efficacy on skin protection [8]. In the present study, aiming at preparing an effective formulation for the treatment of oral cavity diseases, an ethanolic extract of *pompia* rind was loaded in conventional liposomes and liposome-like systems, namely glycerosomes and PG-PEVs (Table 1), which have both been proved to achieve superior performances than conventional liposomes [11,13,15]. The latter were prepared by using different glycerol/water or propylene glycol/water mixtures (12.5%, 25% and 50% *v*/*v*).

**Figure 1:**
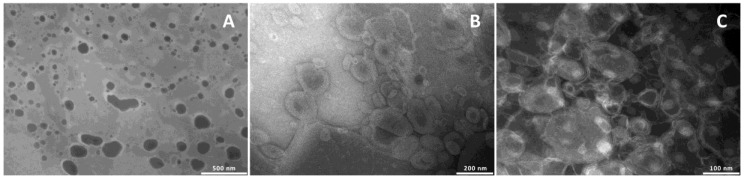
TEM images of *pompia* extract loaded liposomes (**A**), glycerosomes (**B**) and PG-PEVs (**C**).

To confirm the formation of the vesicles and evaluate their structure and size, the formulations were observed by TEM (Figure 1).

Liposomes were unilamellar, and the addition of glycerol or propylene glycol, irrespective of the amount used, led to the formation of multilamellar structures.

The size, polydispersity index, and zeta potential of the *pompia* extract loaded vesicles were estimated and compared with those of empty vesicles in order to evaluate the effect of the incorporation of the extract on vesicle assembly (Table 3). Empty liposomes were small in size (64 nm) and monodispersed (0.25). Similar values were obtained for empty 12glycerosomes, while the use of higher amounts of glycerol (25% and 50%) or propylene glycol (12%, 25% and 50%) led to a slight increase in diameter up to ~100 nm. This is indicative of a marked effect of the glycols on bilayer assembly and structure, which favored the formation of vesicles with a larger curvature radius. As previously found for other extracts [15], the incorporation of the *pompia* extract caused a further increase in size (*p* < 0.05) in all the vesicles. In particular, the mean diameter increased as the glycol amount increased, being ~136 nm for 12glycerosomes and 12PG-PEVs, and 181 nm for 50glycerosomes and 218 nm for 50PG-PEVs. The zeta potential of empty vesicles was highly negative (~−70 mV), and became less negative in extract loaded vesicles (~−40 mV). The extract loaded glycerosomes displayed a narrow size distribution (polydispersity index ≤0.25), while PG-PEVs were more polydispersed, as PI was ≤0.39.

The EE% of the vesicles was around 63%, irrespective of the formulation (Table 3).

Stability studies performed at room temperature for 30 days confirmed the good stability of both glycerosomes and PG-PEVs, since only a slight variation of the mean diameter was detected (~±10%), while a ~±30% variation was found for conventional liposomes. The higher stability of glycerosomes and PG-PEVs may be related to the ability of both glycols to increase the viscosity of the dispersion, which prevented aggregation or fusion phenomena.

### 3.3. In Vitro Antioxidant Activity of the Formulations

To evaluate the protective effect of the *pompia* extract loaded vesicles on buccal mucosa, keratinocytes were used in the in vitro antioxidant tests. The oral mucosa, which acts as a barrier against external environmental aggressors, consists of two distinct layers: the surface stratified epithelium and the deeper fibrous connective tissue (*lamina propria*). The epithelium is composed primarily of keratinocytes (~90%), but also melanocytes, Langerhans & Merkel cells, lymphocytes and macrophages. For this reason, keratinocytes represent the first line of defence against exogenous substances and oxidative stress, and were, therefore, chosen for the in vitro studies.

The keratinocytes were stressed with hydrogen peroxide and simultaneously treated with the formulations. A dispersion of the extract in water was used as a reference.

**Figure 2:**
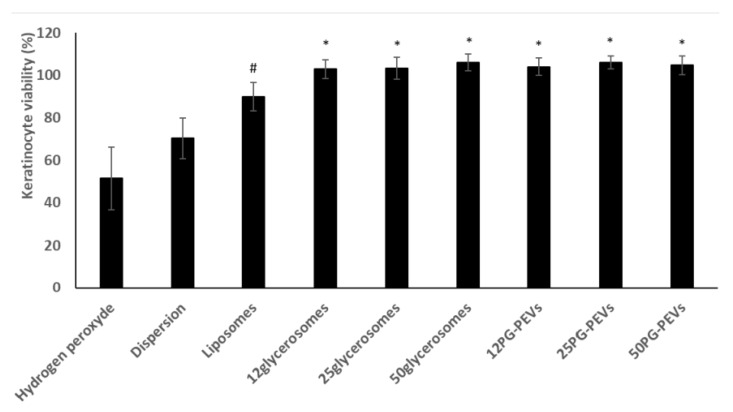
Viability of keratinocytes stressed with hydrogen peroxide and simultaneously treated with the *pompia* extract in dispersion or loaded in the vesicular formulations. Mean values ± standard deviations are reported. ^#^ and * symbols indicate statistical differences between samples.

The viability of keratinocytes treated with H_2_O_2_ decreased significantly to 50% (Figure 2).

The extract dispersion protected the cells from oxidative stress reducing mortality (~75% viability). The incorporation of the extract in liposomes provided a further increase in viability (~90%), which approached 100% when the extract was delivered by glycerosomes and PG-PEVs (Figure 2). Glycerosomes and PG-PEVs displayed the highest ability to counteract the harmful effect of hydrogen peroxide, regardless of the amount and type of glycol used, re-establishing healthy conditions.

### 3.4. In Vitro Antibacterial Activity of the Formulations

The antibacterial activity of the extract loaded liposomes, glycerosomes and PG-PEVs was assessed by measuring the diameter of the bacterial growth inhibition zone (inhibition halo, Table 4).

The extract dispersed in water was used as a reference. The inhibition halo of *S. mutans* and *L. acidophilus* provided by the extract in dispersion was similar to that observed with the application of the vesicles, regardless of which formulation was tested (*p* > 0.05), thus indicating that the antimicrobial capacity of *pompia* extract against the selected cariogenic bacteria was not affected by the incorporation in the vesicles. On the other hand, the extract loaded vesicles increased the aggressiveness of the extract towards the commensal *S. sanguinis:* indeed, the halo inhibition increased from ~8 mm to ~13 mm. Additionally, the use of vesicular formulation may regulate the distribution and release of the active components in the oral cavity.

## 4. Conclusions

The oral cavity is constantly exposed to irritants and pathogens that can cause local disorders associated with oxidative stress and/or microbial infections. Additionally, pathologies such as periodontitis, caries, oral precancerosis, and gingivitis can increase oxidative injury. The use of *pompia* extract loaded glycerosomes and PG-PEVs, which combine the carrier ability of phospholipid vesicles with the penetration enhancing ability of the glycols, may represent an attractive strategy for the protection of the oral cavity against oxidative and bacterial injuries.

## Figures and Tables

**Table 1:** Composition (mg/mL) of liposomes, glycerosomes and propylene glycol-penetration enhancers containing vesicles (PG-PEVs).

Formulation	Extract (mg)	S75 (mg)	Glycerol (mL)	PG (mL)	H_2_O (mL)
Liposomes	80	120	0	0	1.00
12glycerosomes	80	120	0.25	0	1.75
25glycerosomes	80	120	0.50	0	1.50
50glycerosomes	80	120	1.00	0	1.00
12PG-PEVs	80	120	0	0.25	1.75
25PG-PEVs	80	120	0	0.50	1.50
50PG-PEVs	80	120	0	1.00	1.00

S75: Lipoid S75. PG: propylene glycol.

**Table 2:** Main components of the *pompia* extract.

Analyte	µg/mg
Gallic acid	128.3 ± 10.9
Eriocitrin	40.4 ± 2.1
Neoeriocitrin	42.5 ± 3.5
Naringin	28.0 ± 1.6
Hesperidin	16.9 ± 0.9
Neohesperidin	76.5 ± 3.8
Myricetin 3-galactoside	29.3 ± 1.4

**Table 3:** Mean diameter (MD), polydispersity index (PI), zeta potential (ZP) and entrapment efficiency (EE) of empty and *pompia* extract loaded liposomes, glycerosomes and PG-PEVs. Mean values ± standard deviation (SD) were calculated from at least 3 replicates.

Formulation	MD (nm ± SD)	PI	ZP (mV ± SD)	EE (% ± SD)
Empty liposomes	64 ± 7	0.25	−69 ± 4	
Empty 12glycerosomes	65 ± 6	0.25	−78 ± 3	
Empty 25glycerosomes	96 ± 6	0.27	−77 ± 4	
Empty 50glycerosomes	106 ± 6	0.316	−70 ± 3	
Empty 12PG-PEVs	87 ± 78	0.26	−76 ± 6	
Empty 25PG-PEVs	99 ± 8	0.29	−76 ± 4	
Empty 50PG-PEVs	105 ± 7	0.27	−76 ± 5	
*Pompia* liposomes	137 ± 16	0.26	−43 ± 4	59 ± 11
*Pompia* 12glycerosomes	139 ± 2	0.18	−40 ± 3	68 ± 11
*Pompia* 25glycerosomes	157 ± 13	0.22	−40 ± 9	65 ± 9
*Pompia* 50glycerosomes	181 ± 33	0.25	−48 ± 6	66 ± 12
*Pompia* 12PG-PEVs	133 ± 18	0.35	−49 ± 4	63 ± 9
*Pompia* 25PG-PEVs	152 ± 16	0.34	−39 ± 6	61 ± 8
*Pompia* 50PG-PEVs	218 ± 28	0.39	−45 ± 5	64 ± 10

**Table 4:** Inhibition halo (IH) for *pompia* extract in dispersion or loaded in the vesicular formulations against *S. mutans*, *L. acidophilus*, and *S. sanguinis*. Mean values ± standard deviation (SD) are reported.

Formulation	IH (mm ± SD)		
	*S. mutans*	*L. acidophilus*	*S. sanguinis*
*Pompia* dispersion	10 ± 5	10 ± 4	8 ± 4
*Pompia* liposomes	12 ± 5	10 ± 3	13 ± 4
*Pompia* 12glycerosomes	11 ± 5	12 ± 3	12 ± 4
*Pompia* 25glycerosomes	12 ± 5	12 ± 4	13 ± 4
*Pompia* 50glycerosomes	12 ± 4	13 ± 2	12 ± 3
*Pompia* 12PG-PEVs	11 ± 3	12 ± 5	14 ± 3
*Pompia* 25PG-PEVs	12 ± 4	10 ± 4	13 ± 5
*Pompia* 50PG-PEVs	10 ± 5	11 ± 5	14 ± 4
